# Clinicopathologic features and treatment efficacy of Chinese patients with *BRAF*-mutated metastatic colorectal cancer: a retrospective observational study

**DOI:** 10.1186/s40880-017-0247-y

**Published:** 2017-10-16

**Authors:** Xicheng Wang, Qing Wei, Jing Gao, Jian Li, Jie Li, Jifang Gong, Yanyan Li, Lin Shen

**Affiliations:** Department of Gastrointestinal Oncology, Key Laboratory of Carcinogenesis and Translational Research (Ministry of Education), Peking University Cancer Hospital & Institute, Beijing, 100142 P. R. China

**Keywords:** Colorectal cancer, *BRAF*, Chemotherapy, Prognosis

## Abstract

**Background:**

The prognostic role of the V600E mutation of v-raf murine sarcoma viral oncogene homolog B1 (*BRAF*) in metastatic colorectal cancer (mCRC) is well established, but the therapeutic regimen targeting this disease is lacking. This study aimed to analyze the clinicopathologic features of and treatment efficacy of commonly used regimens on *BRAF*-mutated mCRCs.

**Methods:**

We collected and reviewed the medical records of mCRC patients treated at Peking University Cancer Hospital & Institute (Beijing, China) between July 2011 and July 2016. Kirsten rat sarcoma viral oncogene homolog (*KRAS*), neuroblastoma *RAS* viral oncogene homolog (*NRAS*), and *BRAF* mutational status was assayed using direct sequencing. The details of clinicopathologic characteristics of patients and their responses to FOLFOXIRI regimen or standard therapy were obtained by reviewing the medical records. The progression-free survival (PFS) and overall survival (OS) were assessed using Kaplan–Meier analysis and compared using the log-rank test.

**Results:**

Of 1694 patients studied, 75 had *BRAF* exon 15 mutations. Of these 75 patients, 71 had V600E mutation, 1 had D594G mutation, 2 had K601E mutation, and 1 had a novel T599_V600insAGA alteration. No patients had *KRAS* or *NRAS* mutations. Of 63 patients with *BRAF* V600E-mutated mCRC and sufficient clinical data, 27 (42.9%) had right-sided colon tumors, 19 (30.2%) had left-sided colon tumors, and 17 (26.9%) had rectal tumors; 26 (41.3%) had peritoneal metastases, and 50 (79.4%) had distant lymph node metastases. The patients with *BRAF* K601E- and T599_V600insAGA-mutated tumors had similar clinicopathologic features to those with *BRAF* V600E-mutated tumors. Patients with the *BRAF* V600E mutation benefited more from FOLFOXIRI regimen compared with patients who underwent standard therapy (overall response rate 83.3% vs. 14.0%; median PFS 6.4 months vs. 2.8 months, *P* = 0.220; median OS 11.0 months vs. 6.9 months, *P* = 0.048).

**Conclusions:**

*BRAF* V600E mutations were commonly identified in right-sided tumors and showed a high incidence of peritoneal and distant lymph nodes metastases. This subtype of mCRC was characterized by short OS and unique patterns of metastasis. Compared with standard treatment regimens, the FOLFOXIRI regimen had acceptable and manageable toxicities and favorable efficacy on patients with *BRAF*-mutated mCRC.

## Background

V-raf murine sarcoma viral oncogene homolog B1 (*BRAF*), a principal downstream effector of the mitogen-activated protein kinase (*MAPK*)/extracellular signal-regulated kinase (*ERK*) pathway, is mutated in 5%–10% of colorectal cancer (CRC) cases [1]. It was reported that a thymine to adenine single-base change at position 1799 accounts for 90% of *BRAF* mutations [2]. This missense mutation, located in exon 15, results in a change at codon 600 that substitutes glutamine for valine (V600E) [2]. *BRAF*-mutated CRC tends to be mucinous histologically or poorly differentiated [3, 4]. Clinically, *BRAF*-mutated tumors are primarily located on the right side of the colon and are more prevalent in women and elderly [3, 4]. Lymph nodes and the peritoneum are common metastatic sites [3, 4]. The *BRAF* V600E mutation is negatively associated with prognosis in patients with metastatic colorectal cancer (mCRC), distinguishing them as a subgroup that obtains modest benefit from standard treatments [5–7].

Although V600E mutation is the most frequently reported *BRAF* mutation, some CRC have rare *BRAF* mutations [8]. The studies on *BRAF* mutations beyond codon 600 in CRC are increasing; although their functional roles and clinical relevance have been discussed [9–11], the clinicopathologic features and prognoses of the CRC patients with rare *BRAF* mutations are unclear.

Strategies to manage the aggressiveness of *BRAF*-mutated tumor is challenging. The *BRAF* V600E mutation has been identified as a biomarker of resistance to anti-epidermal growth factor receptor (EGFR) monoclonal antibodies [12]. In addition, results from randomized trials revealed that standard first-line doublets, oxaliplatin + fluorouracil/leucovorin (FOLFOX), oxaliplatin + capecitabine (CapeOX) or irinotecan + fluorouracil/leucovorin (FOLFIRI), plus a monoclonal antibody (cetuximab or bevacizumab) achieved unsatisfactory clinical outcomes for overall survival (OS; 4.3–7.1 months) of *BRAF*-mutated mCRC patients [13, 14]. Nevertheless, in a phase II trial, upfront use of FOLFOXIRI (folinic acid, 5-fluorouracil, oxaliplatin, and irinotecan) plus bevacizumab improved clinical outcomes in patients with *BRAF*-mutated mCRC [15]. The patients’ median progression-free survival (PFS) and median OS were 11.8 and 24.1 months, respectively; overall response rate (ORR) and disease control rate (DCR) were 72% and 88%, respectively [15]. These encouraging results bring hope for the management of this particular subtype of CRC [15].

Subsequently, a recent Tribe 3 randomized trial showed that patients with *BRAF*-mutated CRC who were treated with FOLFOXIRI plus bevacizumab had longer overall survival than those treated with FOLFIRI plus bevacizumab (19 vs. 10.7 months) [16, 17]. However, bevacizumab is associated with several toxicities [18, 19] and patients with a history of bleeding, thrombotic disorders, hemoptysis, cerebral vascular accident, severe cardiac disease (ischemic or congestive heart failure), or bowel obstruction are not ideal candidates for bevacizumab therapy [20]. Therefore, the intensive FOLFOXIRI regimen appears to be an alternative strategy for treating patients with *BRAF*-mutated CRC.

In the present study, we retrospectively collected clinical data and tumor samples from patients with *BRAF*-mutated CRC and analyzed their clinicopathologic characteristics. We also investigated the efficacy of FOLFOXIRI compared with standard doublet-agent treatment (FOLFOX/CapeOX or FOLFIRI) for patients with unresectable *BRAF*-mutated mCRC.

## Patients and methods

### Patient population

**Fig. 1:**
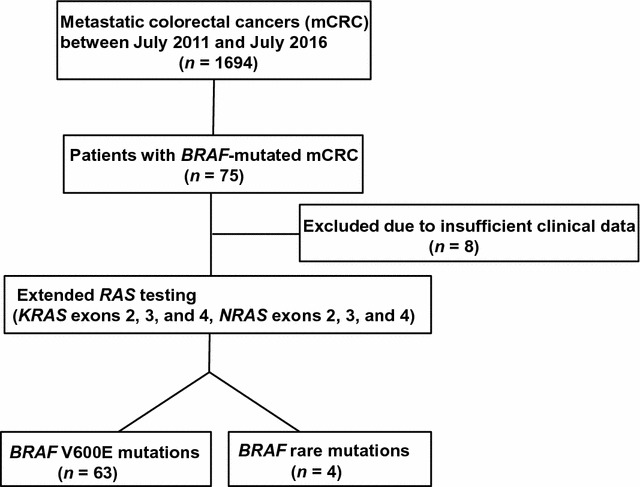
Flow chart of *BRAF* and *RAS* testing procedure. *BRAF* v-raf murine sarcoma viral oncogenes homolog B1; *RAS* rat sarcoma, *NRAS* neuroblastoma *RAS* viral oncogene homolog

All patients with mCRC received *BRAF* (exon 15) testing in Peking University Cancer Hospital & Institute (Beijing, China) between July 2011 and July 2016 (Fig. 1). Patients with *BRAF*-mutated tumors also received extended *RAS* (rat sarcoma) testing including Kirsten rat sarcoma viral oncogene homolog (*KRAS*) exons 2, 3, and 4 and neuroblastoma *RAS* viral oncogene homolog (*NRAS*) exons 2, 3, and 4. The patients with insufficient clinical data were excluded. Clinical parameters, including age, gender, histological diagnosis, Eastern Cooperative Oncology Group performance status (ECOG PS), and tumor anatomic location at the initial presentation, were obtained by reviewing the medical records. Tumor sites were classified as the right-sided colon (including the ileocecal junction, cecum, ascending colon, hepatic flexure, and transverse colon), left-sided colon (including the splenic flexure, descending colon, and sigmoid colon), and rectum. All tumors were staged according to the TNM staging system of the American Joint Committee on Cancer (7th version, 2009). All patients provided written informed consent for use of clinical data and samples in medical research. This study was approved by the Ethics Committee of Peking University Cancer Hospital and performed according to Principles of the Declaration of Helsinki.

### Treatment schedules and evaluation

All patients with *BRAF*-mutated CRC were divided into two groups. Standard therapy group received cytotoxic doublets with or without bevacizumab, and intensive therapy group received a modified FOLFOXIRI regimen. Cytotoxic doublets included CapeOX (oxaliplatin 130 mg/m^2^ intravenous infusion on day 1, capecitabine 1000 mg/m^2^ twice daily per os for 14 days every 3 weeks), mFOLFOX (oxaliplatin 85 mg/m^2^ intravenous infusion on day 1, leucovorin 400 mg/m^2^ intravenous infusion on day 1, fluorouracil 400 mg/m^2^ intravenous injection on day 1, then 2400 mg/m^2^ over 46–48 h continuous infusion every 2 weeks), or FOLFIRI (irinotecan 180 mg/m^2^ intravenous infusion on day 1, leucovorin 400 mg/m^2^ intravenous infusion on day 1, fluorouracil 400 mg/m^2^ intravenous injection day 1, then 2400 mg/m^2^ over 46–48 h continuous intravenous infusion every 2 weeks) at standard doses [21]. Bevacizumab was given at 5 mg/kg intravenous injection every 2 weeks or 7.5 mg/kg intravenous injection every 3 weeks. Treatment was administered until evidence of progression, unacceptable toxicity, or patient refusal or until completion of 12 cycles. Dosing of FOLFOXIRI was as follows: irinotecan 150 mg/m^2^ intravenous infusion on day 1, oxaliplatin 85 mg/m^2^ intravenous infusion on day 1, l-leucovorin (l-LV) 200 mg/m^2^ intravenous infusion on day 1, and 5-fluorouracil (5-FU) 2800 mg/m^2^ as a 48-h continuous intravenous infusion starting on day 1, repeated every 2 weeks for a maximum of 9 cycles. In both groups, treatment modifications were permitted according to adverse events and patients’ tolerance. Adverse events were evaluated according to the National Cancer Institute Common Toxicity Criteria version 3.0. No prophylactic treatment of neutropenia was recommended. Tumor assessment using computed tomography was performed every 6 weeks or when there was evidence of disease progression according to Response Evaluation Criteria in Solid Tumors (RECIST) version 1.1.

### *KRAS*, *NRAS*, and *BRAF* status analysis

Genomic DNA of formalin-fixed, paraffin-embedded (FFPE) sections with ≥ 50% tumor cells (if the content of the tumor cells in sections was lower than 50%, the sections would be microdissected) was extracted using an E.Z.N.A.FFPE DNA Kit (Lot. D3399-01, OMEGA, Norcross, GA, USA) according to the manufacturer’s instructions. All genomic DNA was stored at − 20 °C until use. DNA fragments corresponding to the *KRAS*/*NRAS* gene (exon 2/3/4) and the *BRAF* gene (exon 15) were amplified by PCR using primers shown in Table 1. Each PCR reaction system consisted of 10 × LA PCR buffer II 2 µL, 2.5 mmol/L dNTPs 2 µL, LA Taq 0.1 µL (DRR200A, TaKaRa, Kusatsu, Shiga, Japan), genomic DNA 2 µL, 10 µmol/L forward primer 0.5 µL, and 10 µmol/L reverse primer 0.5 µL in a final volume of 20 µL. The cycling conditions were 95 °C for 5 min; 45 cycles of 95 °C for 30 s, 56 °C for 45 s, and 72 °C for 20 s; and a final extension at 72 °C for 5 min. Details of this type of sequencing are available in the literature [22].

**Table 1:** PCR primers and fragments for *RAS*/*BRAF* testing (*KRAS* exons 2, 3, and 4; *NRAS* exons 2, 3, and 4; and *BRAF* exon 15)

Exon	Primer	Fragment length (bp)
*KRAS*		
Exon 2	F: 5′-TACTGGTGGAGTATTTGATAG-3′	248
	R: 5′-TGGTCCTGCACCAGTAATATG-3′	
Exon 3	F: 5′-GCACTGTAATAATCCAGACTGTG-3′	222
	R: 5′-CCCACCTATAATGGTGAATATCTTC-3′	
Exon 4	F: 5′-ATGACAAAAGTTGTGGACAGGTTTTGA-3′	284
	R: 5′-ATGATTTTGCAGAAAACAGATCTGTATTTATTTCAG-3′	
*NRAS*		
Exon 2	F: 5′-GAACCAAATGGAAGGTCACACT-3′	243
	R: 5′-CCTCACCTCTATGGTGGGATC-3′	
Exon 3	F: 5′-TAGCATTGCATTCCCTGTGGTT-3′	258
	R: 5′-CCTGTAGAGGTTAATATCCGCAA-3′	
Exon 4	F: 5′-GCCACTGTACCCAGCCTAATCTTG-3′	287
	R: 5′-CACATCTCTACCAGAGTTAATCAACTGATGC-3′	
*BRAF*		
Exon 15	F: 5′-CCTAAACTCTTCATAATGCTTGCTC-3′	211
	R: 5′-GTGGAAAAATAGCCTCAATTCTTACC-3′	

*RAS* rat sarcoma, *BRAF* v-raf murine sarcoma viral oncogene homolog B1, *KRAS* Kirsten rat sarcoma viral oncogene homolog, *NRAS* neuroblastoma *RAS* viral oncogene homolog

### Follow-up

The survival data of each patient was obtained by reviewing information from several sources, including clinical records and telephone follow-up. The follow-up was started from each patient’s diagnosis of metastatic disease and carried out every 3 months. The last follow-up was performed in December 2016. Patients whose vital status could not be ascertained were considered to be lost to follow-up. PFS was defined as the time from the date of first treatment to the date of confirmation of disease progression according to RECIST version 1.1, death from any cause, or the last follow-up. OS was defined as the time from the initiation of treatment to the date of death due to any cause or the last follow-up. Patients who were lost during follow-up or those without any event (progression or death) at the last follow-up were censored.

### Statistical analysis

Statistical analyses were performed with SPSS version 13.0 (SPSS, Inc., Chicago, IL, USA). Chi square test or Fisher’s exact test was used to compare frequencies between groups. The independent-sample Student’s *t* test was used to compare differences between groups. The log-rank test was used to compare Kaplan–Meier survival curves. All tests were two-sided, and *P* < 0.05 were considered statistically significant.

## Results

### Analysis of *KRAS*, *NRAS,* and *BRAF* status

A total of 1694 patients with mCRC received *BRAF* (exon 15) testing, and a *BRAF* mutation was confirmed in 75 patients (Fig. 1). Of these 75 patients, none had *RAS* mutations; 71 had *BRAF* V600E mutations, 2 had *BRAF* K601E mutations that had been previously found in CRC cases [8], 1 had a D594G mutation, and 1 had an AGA insertion between *BRAF* codons 599 and 600 (T599_V600insAGA alteration) that was not previously registered in the COSMIC database (http://cancer.sanger.ac.uk/cosmic) [8].

### Clinicopathologic characteristics of patients with *BRAF* V600E-mutated mCRC

A total of 63 patients with *BRAF* V600E mutation who had sufficient clinical data were evaluated (Table 2). At diagnosis, the median age was 54 years (range 24–79 years); 57 (90.5%) patients had an ECOG PS of 0–1, and 6 (9.5%) had an ECOG PS of 2. Of these patients, 27 (42.9%) had right-sided colon tumors, 19 (30.2%) had left-sided colon tumors, and 17 (26.9%) had rectal tumors; 50 (79.4%) had distant lymph node metastases, 29 (46.0%) had liver metastases, 26 (41.3%) had peritoneal metastases, and 12 (19.0%) had lung involvement. Seventeen patients had only one organ involved in metastasis. Twenty-nine patients had poorly differentiated tumors, and 13 had tumors with mucinous or signet-ring histology.

**Table 2:** Clinicopathologic characteristics of 63 metastatic colorectal cancer (mCRC) patients with *BRAF* V600E mutation

Characteristic	No. of patients (%)
Gender	
Male	27 (42.9)
Female	36 (57.1)
ECOG PS	
0	47 (74.6)
1	10 (15.9)
2	6 (9.5)
Primary tumor	
Right-sided colon	27 (42.9)
Left-sided colon	19 (30.2)
Rectum	17 (26.9)
No. of involved organs	
1	17 (27.0)
> 1	46 (73.0)
Liver metastasis	
Yes	29 (46.0)
No	34 (54.0)
Peritoneal metastasis	
Yes	26 (41.3)
No	37 (58.7)
Distant lymph node metastasis	
Yes	50 (79.4)
No	13 (20.6)
Lung metastasis	
Yes	12 (19.0)
No	51 (81.0)
Mucinous or signet-ring cell component	
Yes	13 (20.6)
No	50 (79.4)
Differentiation (adenocarcinoma)^a^	
Well-moderate	31 (51.7)
Poor	29 (48.3)

*ECOG PS* Eastern Cooperative Oncology Group performance status

^a^Three cases were pure signet-ring cell carcinomas or mucinous carcinomas and were excluded here

### Treatment effects and toxicities

Among 63 patients with *BRAF* V600E mutation, 7 did not receive systemic treatment, and only 56 patients were eligible for first-line treatment response assessment. Table 3 shows the clinicopathologic characteristics of the 56 patients in standard therapy group (treated with cytotoxic doublets with or without bevacizumab regimen) and intensive therapy group (treated with modified FOLFOXIRI regimen). The median age of standard therapy group was 48 years (range 35–79 years), and the median age of intensive therapy group was 44.5 years (range 34–63 years). For patients in standard therapy group, 7 had a partial response, 30 had stable disease, and 13 progressed under treatment. For intensive therapy group, 5 had partial response, and 1 attained stable disease. Patients in two groups had ORRs of 14.0% (7/50) and 83.3% (5/6), respectively, and DCRs of 74.0% (37/50) and 100% (6/6), respectively.

**Table 3:** Characteristics of 56 patients with *BRAF* V600E-mutated mCRC in standard therapy group (treated with cytotoxic doublets with or without bevacizumab regimen) and intensive therapy group (treated with modified FOLFOXIRI regimen)

Variable	Standard therapy group (*n* = 50)	Intensive therapy group (*n* = 6)
Gender		
Male	16 (32.0)	4 (66.7)
Female	34 (68.0)	2 (33.3)
ECOG performance status		
0	42 (84.0)	5 (83.3)
1	8 (16.0)	1 (16.7)
2	0 (0)	0 (0)
Primary tumor		
Right	23 (46.0)	4 (66.7)
Left	15 (30.0)	2 (33.3)
Rectum	12 (24.0)	0 (0)
No. of involved organs		
1	13 (26.0)	4 (66.7)
> 1	37 (74.0)	2 (33.3)
Liver metastases		
Yes	23 (46.0)	6 (100)
No	27 (54.0)	0 (0)
Peritoneal metastases		
Yes	18 (36.0)	1 (16.7)
No	32 (64.0)	5 (83.3)
Distant lymph node metastasis		
Yes	38 (76.0)	5 (83.3)
No	12 (24.0)	1 (16.7)
Lung metastasis		
Yes	9 (18.0)	0 (0)
No	41 (82.0)	6 (100)
Mucinous or signet-ring cell components		
Yes	9 (18.0)	1 (16.7)
No	41 (42.0)	5 (83.3)
Differentiation (adenocarcinoma)^a^		
Well-moderate	27 (54.0)	4 (66.7)
Poor	20 (40.0)	2 (33.3)

All values are presented as number of patients followed by percentage in parentheses

*FOLFOXIRI* 5-fluorouracil, leucovorin, oxaliplatin, and irinotecan

^a^Three cases were pure signet-ring cell carcinomas or mucinous carcinomas and were excluded here

**Fig. 2:**
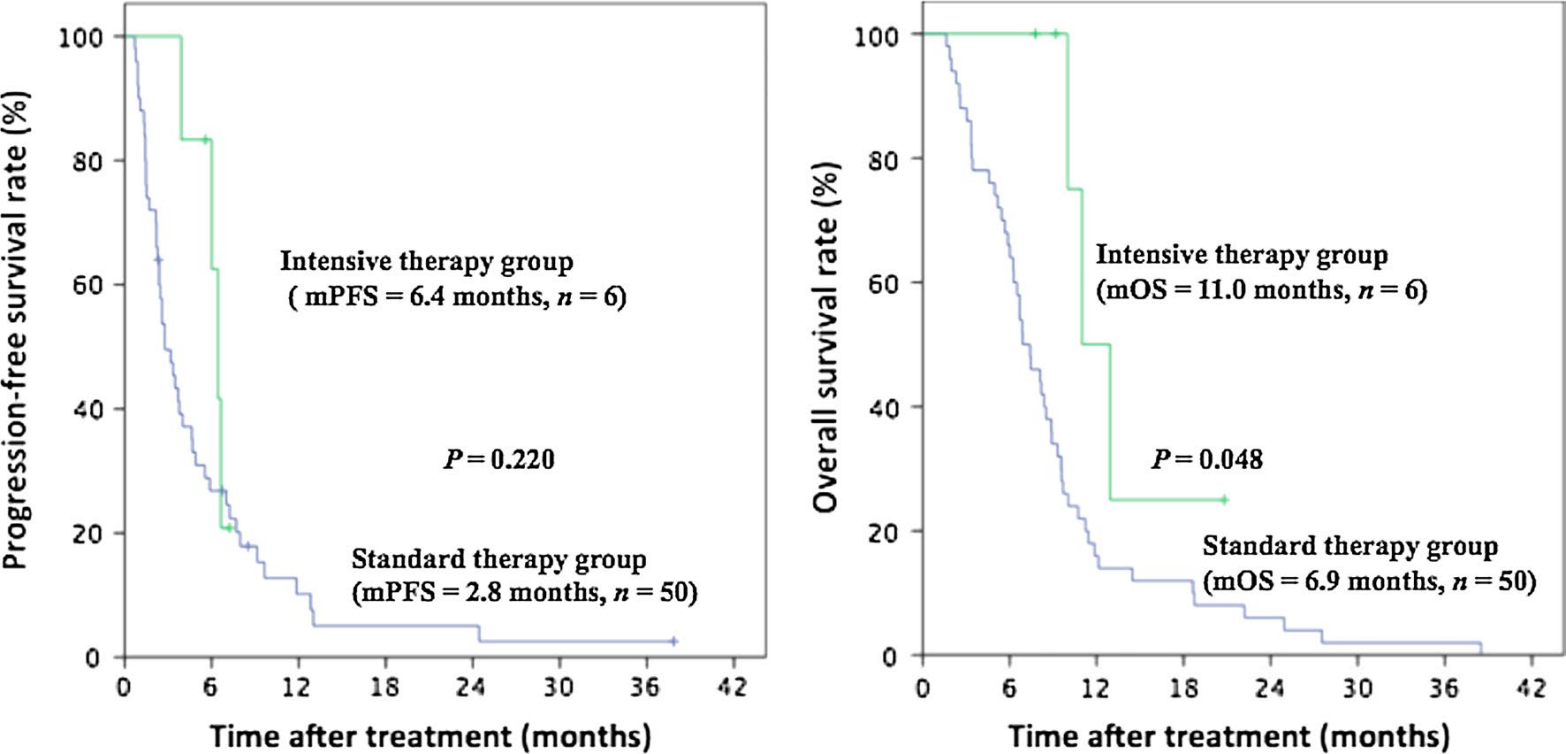
Kaplan–Meier estimates of the survival of standard therapy group (treated with cytotoxic doublets with or without bevacizumab regimen) and intensive therapy group (treated with modified FOLFOXIRI [luorouracil, leucovorin, oxaliplatin, and irinotecan] regimen) among mCRC patients with *BRAF* V600E mutation. *mPFS* median progression-free survival, *mOS* median overall survival

The median follow-up was 11.9 months (95% confidence interval [CI] 7.1–16.2 months). For the 56 patients with *BRAF* V600E mutation, median PFS was 3.7 months (95% CI 2.1–4.8 months), and median OS was 8.1 months (95% CI 6.2–10.0 months). Median PFS was 2.8 months (95% CI 1.8–3.7 months) for standard therapy group and 6.4 months (95% CI 5.5–7.3 months) for intensive therapy group (*P* = 0.220). Median OS was 6.9 months (95% CI 5.3–8.5 months) for standard therapy group and 11.0 months (95% CI 6.3–15.7 months) for intensive therapy group (*P* = 0.048) (Fig. 2).

For the 56 patients assessable for treatment-associated toxicity, both treatments were relatively well tolerated, and the toxicities were manageable (Table 4); no toxicity-related death occurred in both groups. The most common toxicities were neutropenia (48/56, 85.7%), anemia (34/56, 60.7%), peripheral neurotoxicity (30/56, 53.6%), nausea (27/56, 48.2%), astenia (24/56, 42.8%), thrombocytopenia (20/56, 35.7%), diarrhea (17/56, 30.4%), and vomiting (17/56, 30.4%). The only case of febrile neutropenia occurred in standard therapy group (1/56, 1.8%).

**Table 4:** The most common toxicities in patients with *BRAF* V600E-mutated mCRC in standard therapy group (treated with cytotoxic doublets with or without bevacizumab) and intensive therapy group (treated with modified FOLFOXIRI regimen)

Toxicity (NCI-CTC grade)	Standard therapy group (*n* = 50)				Intensive therapy group (*n* = 6)			
	Grade 1	Grade 2	Grade 3	Grade 4	Grade 1	Grade 2	Grade 3	Grade 4
Thrombocytopenia	18 (36.0)	1 (2.0)	0 (0)	0 (0)	0 (0)	1 (16.7)	0 (0)	0 (0)
Anemia	15 (30.0)	14 (28.0)	3 (6.0)	0 (0)	0 (0)	2 (33.3)	0 (0)	0 (0)
Nausea	15 (30.0)	9 (18.0)	0 (0)	0 (0)	2 (33.3)	1 (16.7)	0 (0)	0 (0)
Peripheral neurotoxicity	13 (26.0)	14 (28.0)	0 (0)	0 (0)	2 (33.3)	1 (16.7)	0 (0)	0 (0)
Astenia	11 (22.0)	11 (22.0)	1 (2.0)	0 (0)	0 (0)	1 (16.7)	0 (0)	0 (0)
Vomiting	10 (20.0)	5 (10.0)	0 (0)	0 (0)	2 (33.3)	0 (0)	0 (0)	0 (0)
Diarrhea	10 (20.0)	4 (8.0)	0 (0)	0 (0)	2 (33.3)	1 (16.7)	0 (0)	0 (0)
Neutropenia	10 (20.0)	13 (26.0)	15 (30.0)	6 (12.0)	0 (0)	2 (33.3)	1 (16.7)	1 (16.7)
Stomatitis	9 (18.0)	5 (10.0)	0 (0)	0 (0)	1 (16.7)	0 (0)	0 (0)	0 (0)
Hypertension	3 (6.0)	0 (0)	0 (0)	0 (0)	0 (0)	0 (0)	0 (0)	0 (0)
Hemorrhage	3 (6.0)	0 (0)	0 (0)	0 (0)	0 (0)	0 (0)	0 (0)	0 (0)

All values are presented as number of patients followed by percentage in parentheses

*FOLFOXIRI* 5-fluorouracil, leucovorin, oxaliplatin, and irinotecan, *NCI-CTC* National Cancer Institute Common Toxicity Criteria

### Rare *BRAF* mutations

Four patients who had right-sided colon tumors with rare *BRAF* mutations were identified (Table 5). All 4 patients had liver metastases, whereas none of them had lung metastasis. The mutations were *BRAF* T599_V600insAGA in 1 patient and K601E in 2 patients. These 3 patients had peritoneal and distant lymph node metastasis with mucinous pathology. The fourth patient carried a D594G mutation. None of the 4 mCRC patients harboring rare *BRAF* mutations reached any therapeutic endpoint yet. At the time of data cutoff on December 2016, patient 4 did not progress after 9 months of treatment.

**Table 5:** Clinicopathologic characteristics of 4 mCRC patients with rare *BRAF* mutations

Patient	Gender	*BRAF* mutation	Age (years)	TNM stage	Differentiation	Mucinous components	Peritoneal metastases	Distant lymph node metastasis
Patient 1	Female	T599_V600insAGA	63	T4aN1cM1	Moderate	No	Yes	Yes
Patient 2	Female	K601E	65	T4N2M1	Moderate	Yes	Yes	Yes
Patient 3	Male	K601E	42	T4N2M1	Poor	Yes	Yes	Yes
Patient 4	Female	D594G	55	T3N2M1	Moderate	No	No	No

All of the 4 patients harboring rare *BRAF* mutations were identified with right-sided tumors and showed liver metastases

None of the 4 patients showed lung metastases

## Discussion

In the present study, *BRAF* V600E-mutated tumors were more frequently located on the right-sided colon (27/63, 42.9%); nearly half of the patients (26/63, 41.3%) had peritoneal metastasis. mCRC with *BRAF* V600E mutation was more likely to metastasize to distant lymph nodes (79.4%, 50/63). The median age at diagnosis was 54 years. The median PFS of patients with *BRAF* V600E mutation was 3.7 months, and median OS was 8.1 months. Thus, this disease was likely to be associated with poor prognosis.

The high occurrence rates of peritoneal and distant lymph node metastases were consistent with the results of previous reports [3, 23, 24]. Tran et al. [23] reported peritoneal metastases in 46% and distant lymph node metastases in 53% of *BRAF*-mutated mCRCs. Earlier studies identified an association between *BRAF* mutations in mCRC and older age; the patients with *BRAF*-mutated mCRC were significantly elder than those with *BRAF* wild-type tumors (median age 64 years vs. 58 years; *P* < 0.01) [3]. In contrast, patients with *BRAF* mutations in our study were younger, with a median age of 54 years. Western patients with *BRAF* V600E-mutated tumors showed poor outcome with a median survival of 7.2 months [24], which is comparable to the OS of 8.1 months in our study. We confirmed that *BRAF* V600E-mutated tumors represent a discrete subset of CRC characterized by short OS and unique patterns of metastasis.

Additionally, early work suggested that monotherapy with *BRAF* inhibitor vemurafenib or another single *BRAF* inhibitor did not produce the desired antitumoral activity and clinical efficacy [25, 26]. Yaeger et al. [27] reported that median PFS for the patients with *BRAF*-mutated mCRC who received vemurafenib combined with the anti-EGFR antibody panitumumab was 3.2 months and median OS was 7.6 months. Even a doublet cytotoxic regimen offered modest clinical activity against this highly aggressive and chemoresistant subset of CRC. To date, no effective strategies have been developed to counteract the aggressiveness of *BRAF*-mutated tumors. Our data suggest that a FOLFOXIR regimen may be effective in such cases. The FOLFOXIRI regimen prolonged PFS and OS, although these outcomes were less favorable than those reported in previous studies [15, 16]. Most of our patients had more than one metastatic site and the sample size was small, which may limit our analysis of survival benefits derived from a triplet-agent treatment. Moreover, the FOLFOXIRI regimen had acceptable and manageable toxicities compared with standard treatment.

In addition, we found K601E mutations in 2 patients diagnosed with advanced-stage CRC and tumors on the right side of the colon who had metastases to distant lymph nodes and the peritoneum. Finally, the *BRAF* D594G mutation has been previously reported but data suggested that it may not have an association with aggressive tumor phenotypes. Amaki-Takao et al. [10] reported that patients with *BRAF* D594G-mutated CRCs had similar clinicopathologic features and prognosis as those with *BRAF* wild-type CRCs. However, only one patient in the present study had *BRAF* D594G mutation. This patient had stable disease until the last follow-up and no progression occurred within 9 months after first-line treatment. In addition, one novel *BRAF* T599_V600insAGA alteration was found that has not yet been described previously [8]. The patient was a 63-year-old woman with a primary tumor on the right side of the colon, and she also had lymph node and peritoneal metastases. Based on patient clinical characteristics and the insertion next to the V600 position on the *BRAF*, we suspect that this newly discovered mutation might have a similar prognostic role as the V600E mutation.

Several patients with *BRAF* mutation in the present study had longer survival than others, and the data suggest that heterogeneity within *BRAF* V600E mutations may explain this variation. Barras et al. [28] segregated *BRAF* mutations into two subtypes according to expression of 476 genes and concluded that the patients with each subtype mutation had different OS and relapse-free survival although not significantly. Thus, the molecular landscapes in 63 patients with *BRAF* V600E-mutated tumors in the present study would be of interest but are yet to be determined. Also, the effect of a novel *BRAF* alteration (T599_V600insAGA) on prognosis and in vivo functional testing for this mutation warrants further investigation.

The present study had some limitations. First, there was possible selection bias caused by the retrospective nature of the study and a single cancer. Second, we did not analyze microsatellite stability status and how it influences *BRAF* status. Third, few patients were treated with the FOLFOXIRI regimen, limiting analysis of survival benefit from this intensive regimen. Even so, this study is a relative large-size report evaluating the *BRAF* mutation in Chinese patients with mCRC.

In conclusion, *BRAF* V600E mutations were commonly identified in right-sided mCRCs. High incidences of peritoneal and distant lymph node metastases were observed in mCRC with *BRAF* V600E mutations. In spite of the poor prognosis, the FOLFOXIRI regimen have shown more favorable efficacy on patients with *BRAF*-mutated mCRC with acceptable and manageable toxicities compared with standard treatment regimens.

## References

[1] Tol J, Nagtegaal I (2009). Punt C.BRAF mutation in metastatic colorectal cancer. N Engl J Med.

[2] Cantwell-Dorris ER, O’Leary JJ, Sheils OM.BRAF V600E: implications for carcinogenesis and molecular therapy. Mol Cancer Ther. 2011;10(3):385–94. 10.1158/1535-7163.MCT-10-0799 21388974

[3] Yaeger R, Cercek A, Chou JF, Sylvester BE, Kemeny NE, Hechtman JF (2014). BRAF mutation predicts for poor outcomes after metastasectomy in patients with metastatic colorectal cancer. Cancer.

[4] Samowitz WS, Sweeney C, Herrick J, Albertsen H, Levin TR, Murtaugh MA (2005). Poor survival associated with the BRAF V600E mutation in microsatellite-stable colon cancers. Cancer Res.

[5] Richman SD, Seymour MT, Chambers P, Elliott F, Daly CL, Meade AM (2009). KRAS and BRAF mutations in advanced colorectal cancer are associated with poor prognosis but do not preclude benefit from oxaliplatin or irinotecan: results from the MRC FOCUS trial. J Clin Oncol.

[6] Heinemann V, von Weikersthal LF, Decker T, Kiani A, Vehling-Kaiser U, Al-Batran SE (2014). FOLFIRI plus cetuximab versus FOLFIRI plus bevacizumab as first-line treatment for patients with metastatic colorectal cancer (FIRE-3): a randomised, open-label, phase 3 trial. Lancet Oncol..

[7] Douillard JY, Oliner KS, Siena S, Tabernero J, Burkes R, Barugel M (2013). Panitumumab-FOLFOX4 treatment and RAS mutations in colorectal cancer. N Engl J Med.

[8] COSMIC: Catalogue of Somatic Mutation in Cancer. Gene variants for BRAF. http://cancer.sanger.ac.uk/cosmic/gene/analysis?ln=BRAF Accessed 31 Mar 2017.

[9] Mori Y, Nagasaka T, Mishima H, Umeda Y, Inada R, Kishimoto H (2015). The rare BRAF VK600-601E mutation as a possible indicator of poor prognosis in rectal carcinoma—a report of a case. BMC Med Genet.

[10] Amaki-Takao M, Yamaguchi T, Natsume S, Iijima T, Wakaume R, Takahashi K (2016). Colorectal cancer with BRAF D594G mutation is not associated with microsatellite instability or poor prognosis. Oncology..

[11] Cremolini C, Bartolomeo MD, Amatu A, Antoniotti C, Moretto R, Berenato R (2015). BRAF codons 594 and 596 mutations identify a new molecular subtype of metastatic colorectal cancer at favorable prognosis. Ann Oncol.

[12] Van Cutsem E, Cervantes A, Adam R, Sobrero A, Van Krieken JH, Aderka D (2016). ESMO consensus guidelines for the management of patients with metastatic colorectal cancer. Ann Oncol..

[13] Souglakos J, Philips J, Wang R, Marwah S, Silver M, Tzardi M (2009). Prognostic and predictive value of common mutations for treatment response and survival in patients with metastatic colorectal cancer. Br J Cancer.

[14] Bokemeyer C, Van Cutsem E, Rougier P, Ciardiello F, Heeger S, Schlichting M (2012). Addition of cetuximab to chemotherapy as first-line treatment for KRAS wild-type metastatic colorectal cancer: pooled analysis of the CRYSTAL and OPUS randomised clinical trials. Eur J Cancer.

[15] Loupakis F, Cremolini C, Salvatore L, Masi G, Sensi E, Schirripa M (2014). FOLFOXIRI plus bevacizumab as first-line treatment in BRAF, mutant metastatic colorectal cancer. Eur J Cancer.

[16] Cremolini C, Loupakis F, Antoniotti C, Lupi C, Sensi E, Lonardi S (2015). FOLFOXIRI plus bevacizumab versus FOLFIRI plus bevacizumab as first-line treatment of patients with metastatic colorectal cancer: updated overall survival and molecular subgroup analyses of the open-label, phase 3 TRIBE study. Lancet Oncol..

[17] Mellas N, Benbrahim Z, El Mesbahi O (2014). Colorectal cancer: new developments after the 2013 ECCO/ESMO congress. Chin J Cancer..

[18] Hurwitz H, Fehrenbacher L, Novotny W, Cartwright T, Hainsworth J, Heim W (2004). Bevacizumab plus irinotecan, fluorouracil, and leucovorin for metastatic colorectal cancer. N Engl J Med.

[19] Giantonio BJ, Catalano PJ, Meropol NJ, O’Dwyer PJ, Mitchell EP, Alberts SR (2007). Bevacizumab in combination with oxaliplatin, fluorouracil, and leucovorin (FOLFOX4) for previously treated metastatic colorectal cancer: results from the Eastern Cooperative Oncology Group Study E3200. J Clin Oncol.

[20] Caprioni F, Fornarini G (2007). Bevacizumab in the treatment of metastatic colorectal cancer. Future Oncol..

[21] National Comprehensive Cancer Network. NCCN clinical practice guidelines in Oncology: colon cancer (2017.V1)[EB/OL]. (2016-11-23) [2016-12-22]. https://www.nccn.org/professionals/physician_gls/f_guidelines.asp.

[22] Wei Q, Wang X, Gao J, Li J, Li J, Qi C (2016). Clinicopathologic and molecular features of colorectal adenocarcinoma with signet-ring cell component. PLoS ONE.

[23] Tran B, Kopetz S, Tie J, Gibbs P, Jiang ZQ, Lieu CH (2011). Impact of BRAF mutation and microsatellite instability on the pattern of metastatic spread and prognosis in metastatic colorectal cancer. Cancer.

[24] Tie J, Gibbs P, Lipton L, Christie M, Jorissen RN, Burgess AW (2011). Optimizing targeted therapeutic development: analysis of a colorectal cancer patient population with the BRAF(V600E) mutation. Int J Cancer.

[25] Mao M, Tian F, Mariadason JT, Tsao CC, Lemos RJ, Dayyani F (2013). Resistance to BRAF inhibition in BRAF-mutant colon cancer can be overcome with PI3K inhibition or demethylating agents. Clin Cancer Res.

[26] Coffee EM, Faber AC, Roper J, Sinnamon MJ, Goel G, Keung L (2013). Concomitant BRAF and PI3K/mTOR blockade is required for effective treatment of BRAFV600E colorectal cancer. Clin Cancer Res.

[27] Yaeger R, Cercek A, O’Reilly EM, Reidy DL, Kemeny N, Wolinsky T (2015). Pilot trial of combined BRAF and EGFR inhibition in BRAF-mutant metastatic colorectal cancer patients. Clin Cancer Res.

[28] Barras D, Missiaglia E, Wirapati P, Sieber OM, Jorissen RN, Love C (2017). BRAF V600E mutant colorectal cancer subtypes based on gene expression. Clin Cancer Res.

